# Ethanolamine enhances adhesion, promotes microcompartment formation, and modulates gene expression in *Levilactobacillus brevis* ATCC 14869

**DOI:** 10.1080/19490976.2024.2350778

**Published:** 2024-05-08

**Authors:** Polycronis P. Akouris, Gerrit A. Stuivenberg, John A. Chmiel, Wongsakorn Kiattiburut, Annabel Poon, Gregor Reid, Jeremy P. Burton

**Affiliations:** a Temerty Faculty of Medicine, University of Toronto, Toronto, ON, Canada; b Canadian Centre for Human Microbiome and Probiotics, Lawson Health Research Institute, London, ON, Canada; c Department of Microbiology and Immunology, Western University, London, ON, Canada; d Department of Surgery, Division of Urology, Western University, London, ON, Canada

**Keywords:** Ethanolamine, commensal, microbiota, gene expression, catabolism, adhesion, competitive exclusion

## Abstract

Ethanolamine is an abundant compound in the gastrointestinal tract and a valuable source of carbon and nitrogen for pathogenic bacteria harboring ethanolamine utilization (*eut*) genes. *Eut*-positive pathogens can consume free ethanolamine to outcompete commensal microbes, which often lack *eut* genes, and establish infection. Ethanolamine can also act as a host recognition signal for *eut*-positive pathogens to upregulate virulence genes during colonization. Therefore, reducing free ethanolamine titers may represent a novel approach to preventing infection by *eut*-positive pathogens. Interestingly, the commensal microorganism *Levilactobacillus brevis* ATCC 14869 was found to encode over 18 *eut* genes within its genome. This led us to hypothesize that *L. brevis* can compete with *eut*-positive pathogens by clearing free ethanolamine from the environment. Our results demonstrate that despite being unable to metabolize ethanolamine under most conditions, *L. brevis* ATCC 14869 responds to the compound by increasing the expression of genes encoding proteins involved in microcompartment formation and adhesion to the intestinal epithelial barrier. The improved intestinal adhesion of *L. brevis* in the presence of ethanolamine also enhanced the exclusion of *eut*-positive pathogens from adhering to intestinal epithelial cells. These findings support further studies to test whether *L. brevis* ATCC 14869 can counter enteric pathogens and prevent or reduce the severity of infections. Overall, the metabolic capabilities of *L. brevis* ATCC 14869 offer a unique opportunity to add to the armamentarium of antimicrobial therapies as well as our understanding of the mechanisms used by beneficial microbes to sense and adapt to host microenvironments.

## Introduction

Ethanolamine (H_2_N-CH_2_-CH_2_-OH) is a constituent of phosphatidylethanolamine, which is an essential compound found in all cell membranes.^1^ While titers range from 2 to 46 µM throughout the human body, ethanolamine is particularly abundant in the gastrointestinal tract (up to 5 mM).^2^ This is due to the breakdown of phosphatidylethanolamine through the rapid turnover of cells that make up both the intestinal epithelium and gut microbiota.^3,4^ As such, gut bacteria that possess the *e*thanolamine *ut*ilization (*eut*) operon, which includes the genes to sense, transport, and catabolize ethanolamine, have a significant advantage in this setting.^2^ The *eut* genes are most commonly associated with enteric pathogens that utilize ethanolamine to circumvent nutritional immunity and leverage their growth to establish an infection.^5^

While ethanolamine is an important nutrient source, it also has a significant role in promoting bacterial pathogenesis. It is not uncommon for bacteria to rely on environmental cues to regulate the expression of important genes needed in a particular setting. Certainly, ethanolamine serves the same function and, for *eut*-positive pathogens, upregulates the expression of virulence genes that help these pathogens outcompete the host microbiota to establish a successful infection. For instance, ethanolamine promotes the expression of the genes responsible for cellular adhesion and intracellular survival located outside of the *eut* loci in enterohemorrhagic *E. coli* (EHEC),^6-10^ uropathogenic *E. coli* (UPEC),^11,12^ and *Salmonella enterica* serovar Typhimurium.^12-18^

Despite the prevalence of *eut* genes in enteric pathogens, a small proportion of commensal lactic acid bacteria have also acquired these genes. Of particular interest, *Levilactobacillus brevis* ATCC 14869 possesses over 18 *eut* genes. This includes the enzymatic and structural proteins needed for microcompartment-mediated ethanolamine metabolism. To our knowledge, no lactobacilli harboring these genes have been explored experimentally. Since most ethanolamine-utilizing pathogens rely on this metabolite for both signaling and the circumvention of nutritional immunity, *eut*-positive probiotic strains could potentially mitigate infections by clearing free ethanolamine from the environment. Additionally, these beneficial microbes could also use this compound as a signal to enhance host colonization by upregulating adhesin genes located outside of the *eut* locus. If true, such probiotic microbes could illicit a multifaceted attack by both sequestering nutrients from *eut*-positive pathogens and outcompeting them for space on intestinal epithelia.

In the present study, we sought to determine if ethanolamine is used by *L. brevis* as a nutrient source or a signal to alter adherence capabilities. By combining culture-based techniques and high-performance liquid chromatography (HPLC), the effect of ethanolamine on the growth of *L. brevis* was assessed, while transmission electron microscopy (TEM), RNA sequencing, and qPCR analysis were used to evaluate how ethanolamine impacts *L. brevis* gene expression and bacterial microcompartment formation. Intestinal adherence of *L. brevis* and its ability to competitively exclude pathogen colonization were also assessed using cell culture.

## Results

### Increasing concentrations of ethanolamine do not confer a growth advantage to *L.* *brevis* ATCC 14869

**Figure 1.**
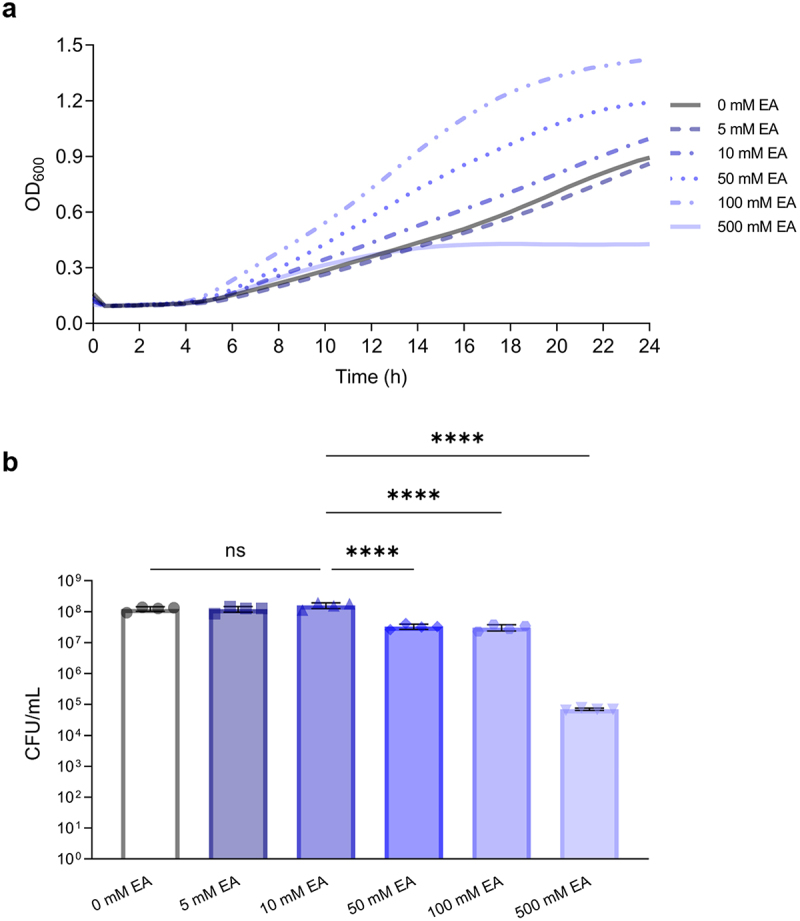
Increasing concentrations of ethanolamine (EA) do not confer a growth advantage to *L. brevis*.

The growth of *L. brevis* in 1:9 diluted MRS supplemented with increasing concentrations of ethanolamine (Figure 1) was assessed. Overall, there was a significant growth difference observed when *L. brevis* was grown at varying ethanolamine titers (ANOVA; *F* = 41.92, *p* < .0001; Figure 1b). At 5 mM (Tukey’s multiple-comparisons test; *p* > .9999) or 10 mM (Tukey’s multiple-comparisons test; *p* = .1504) of ethanolamine there was no significant difference in growth compared to the absence of ethanolamine. Indeed, as ethanolamine concentrations increased to 50 mM, 100 mM and 500 mM, there was a significant decrease in *L. brevis* growth when compared to growth at 10 mM (Tukey’s multiple-comparisons test; *p* < .0001; Figure 1b). The opposite trend was observed when growth was assessed with optical density. Final OD_600_ values were boosted with increasing concentrations of ethanolamine (Figure 1a).

(a) Growth curves of *L. brevis* ATCC 14869 in 1:9 diluted MRS with 150 nM adenosylcobalamin and various concentrations of EA. OD_600_ was measured spectrophotometrically. (b) Bacterial growth was also measured through quantification of CFU/mL after 24 hours. Error bars represent mean ± standard deviation (SD) for 4 biological replicates (one-way ANOVA complemented with Tukey’s multiple-comparison test). ****, *P* < .0001; ns = not significant.

### *Levilactobacillus brevis* ATCC 14869 does not utilize ethanolamine under standard growth conditions in vitro

**Figure 2.**
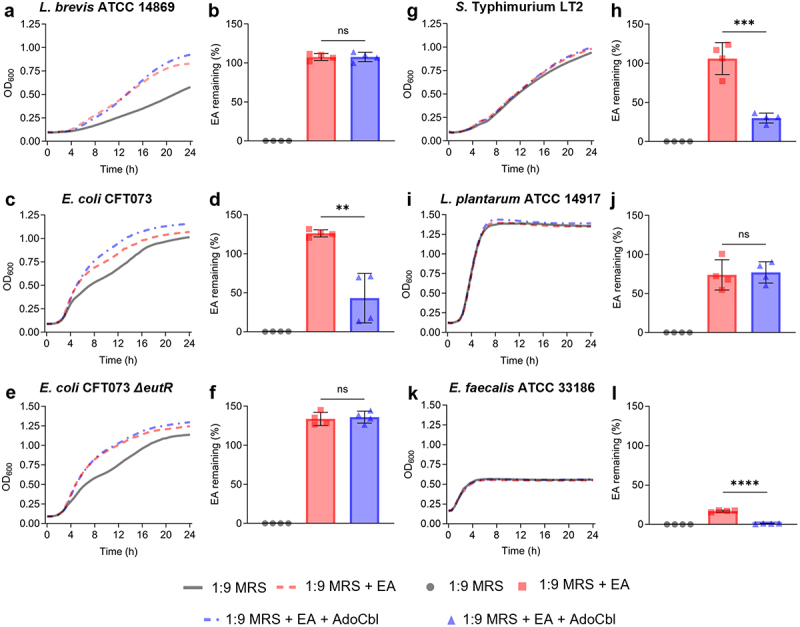
Ethanolamine (EA) clearance during growth.

The bacterium was cultured in 1:9 diluted MRS spiked with 10 mM ethanolamine, with or without 150 nM adenosylcobalamin. Since adenosylcobalamin is a required cofactor for ethanolamine metabolism, final supernatant titers of ethanolamine were compared between the cultures grown with only ethanolamine and those with ethanolamine and adenosylcobalamin. Although there was an increase in OD_600_ measurements (Figure 2a), *L. brevis* ATCC 14869 did not significantly reduce ethanolamine in the supernatant (two-tailed *t* test; *p* = .9656; Figure 2b). In contrast, *E. coli* CFT073 (two-tailed *t* test; *p* = .0020; Figure 2c–d) and *E. faecalis* ATCC 33186 (two-tailed *t* test; *p* < .0001; Figure 2k–l) did not demonstrate an increase in optical density but did significantly reduce ethanolamine in the supernatant when grown in the same conditions. The *E. coli* CFT073 ∆*eutR* mutant showed the same trend in optical density (Figure 2e) but did not significantly reduce ethanolamine titers (two-tailed *t* test; *p* = .7156; Figure 2f). *Salmonella* Typhimurium LT2 was like *E. coli* CFT073, with minimal differences in optical density (Figure 2g) paired with a significant reduction in remaining ethanolamine (two-tailed *t* test; *p* = .004; Figure 2h). *Lactiplantibacillus plantarum*, which is closely related to *L. brevis* genetically but does not possess any *eut* genes, showed no change in optical density (Figure 2i) and did not reduce the ethanolamine spiked into the culture medium (two-tailed *t* test; *p* = .7997; Figure 2j).

All bacteria were grown in 1:9 diluted MRS with or without 10 mM EA and 150 nM adenosylcobalamin (AdoCbl). OD_600_ was monitored spectrophotometrically and remaining EA in the culture supernatant was quantified via HPLC analysis. (a–b) *L. brevis* ATCC 14869. (c–d) *E. coli* CFT073. (e–f) *E. coli* CFT073 *∆eutR*. (g–h) *Salmonella enterica* ssp. enterica serovar Typhimurium LT2. (i–j) *L. plantarum* ATCC 14917. (k–l) *E. faecalis* ATCC 33186. Error bars represent mean ± standard deviation (SD) for 4 biological replicates (unpaired, two-tailed *t* test). **, *P* < .01; ***, *P* < .001; ****, *P* < .0001; ns = not significant.

### Utilization of ethanolamine by *L.* *brevis* ATCC 14869 in reduced nitrogen conditions hampers growth

**Figure 3.**
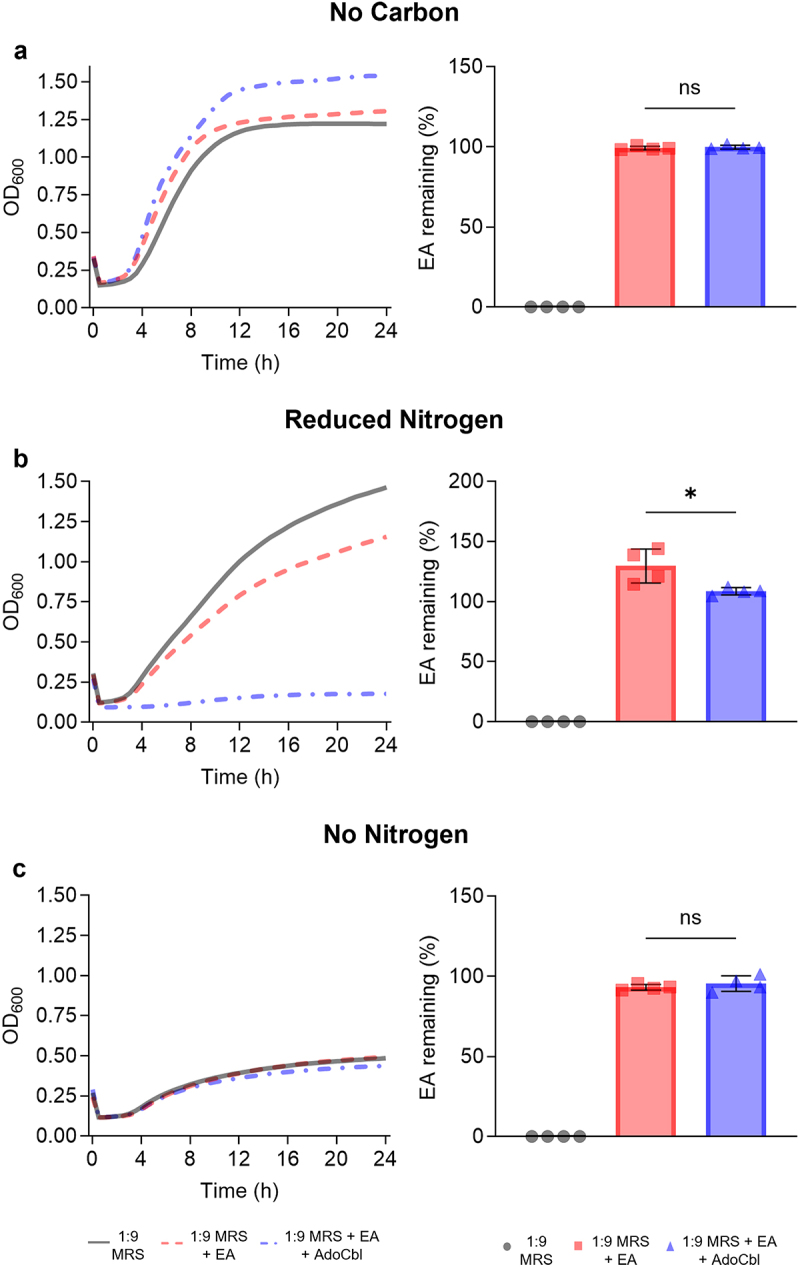
Utilization of ethanolamine as a nitrogen source reduces the growth of *L*. *brevis* ATCC 14869.

The bacterium was cultured in carbon free MRS (cfMRS), reduced nitrogen MRS (rnMRS), or nitrogen free MRS (nfMRS) spiked with 10 mM EA, with or without 150 nM adenosylcobalamin. When grown in cfMRS increased OD_600_ measurements were observed (Figure 3a), however, *L. brevis* ATCC 14869 did not significantly reduce ethanolamine in the supernatant (two-tailed *t* test; *p* = .4941; Figure 3a). Interestingly, when grown in rnMRS, the opposite trend was observed wherein *L. brevis* ATCC 14869 significantly reduced ethanolamine in the supernatant (two-tailed *t* test; *p* = .0270; Figure 3b) which resulted in lower OD_600_ readings. nfMRS seemed insufficient to support substantial growth of the bacterium whether ethanolamine was present or not, as mean OD_600_ did not exceed 0.5 (Figure 3b). In line with these findings, *L. brevis* ATCC 14869 could not reduce the ethanolamine spiked into nfMRS (two-tailed *t* test; *p* = .4131; Figure 3c).

*L*. *brevis* ATCC 14869 was grown in (a–b) cfMRS, (c–d) rnMRS and (e–f) nfMRS with or without 10 mM EA and 150 nM AdoCbl. OD_600_ was monitored spectrophotometrically and remaining EA in the culture supernatant was quantified via HPLC analysis. Error bars represent mean ± standard deviation (SD) for 4 biological replicates (unpaired, two-tailed *t* test). *, *P* < .05; ns = not significant.

### The presence of ethanolamine and adenosylcobalamin results in bacterial microcompartment formation in *L.* *brevis* ATCC 14869

**Figure 4.**
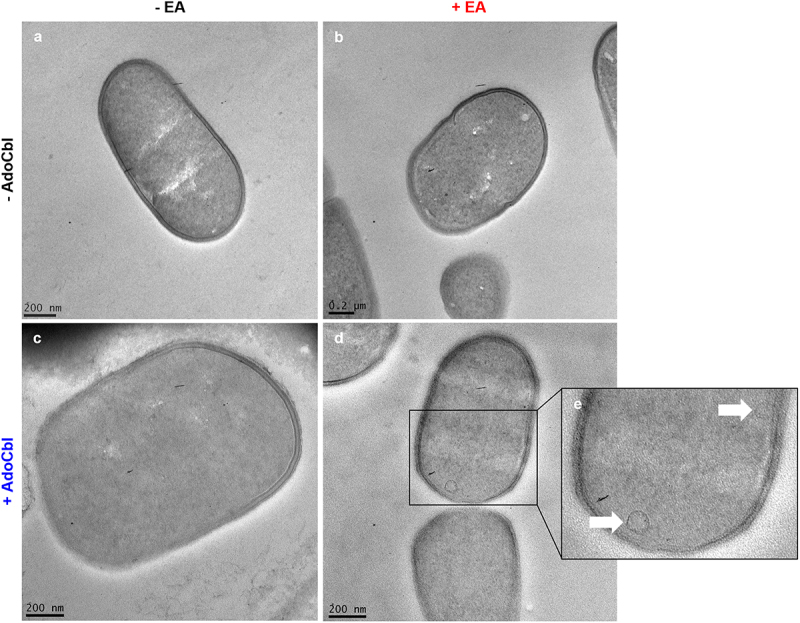
Transmission electron microscopy (TEM) of *L. brevis* ATCC 14869 reveals bacterial microcompartment formation.

Cultures of *L. brevis* ATCC 14869 grown with either 10 mM ethanolamine or 150 nM adenosylcobalamin did not produce microcompartment structures, as determined by TEM (Figure 4a–c). Conversely, bacterial cells grown with both ethanolamine and adenosylcobalamin produced observable microcompartment structures (indicated by white arrows) located near the cellular membrane (Figure 4d–e).

For TEM, cultures of *L. brevis* were grown in 1:9 diluted MRS (a) with 10 mM ethanolamine (EA; b), 150 nM adenosylcobalamin (AdoCbl; c) or both metabolites (d–e). After 24 hours of growth, bacterial cells were fixed and embedded prior to sectioning and contrast staining to qualitatively observe the presence of microcompartment structures within individual cells.

### Ethanolamine and adenosylcobalamin upregulate *eut*, *pdu*, and *apf* gene expression in *L.* *brevis* ATCC 14869

**Figure 5.**
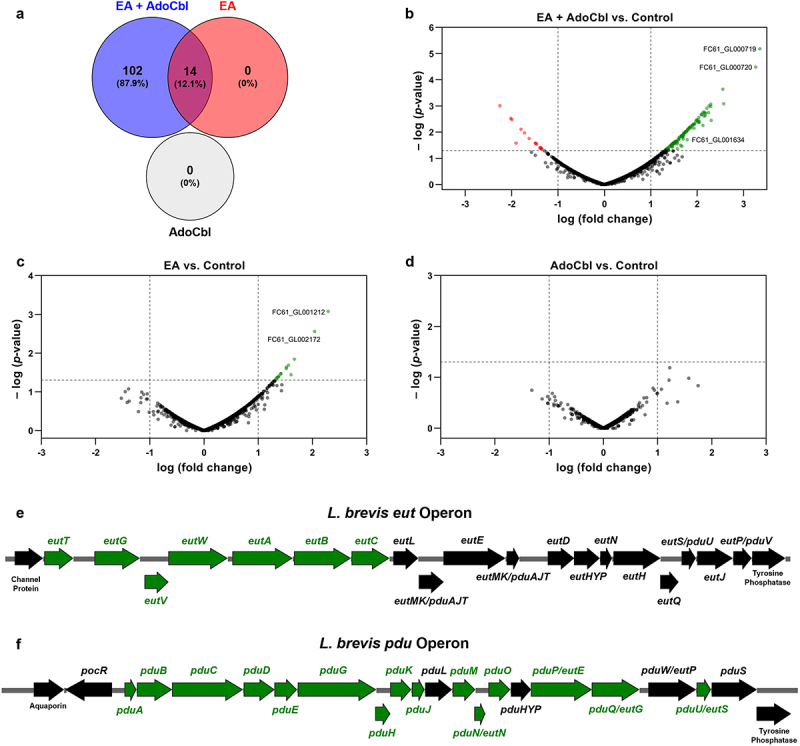
RNA sequencing analysis of *L. brevis* ATCC 14869 grown with ethanolamine (EA) and adenosylcobalamin (AdoCbl).

**Figure 6.**
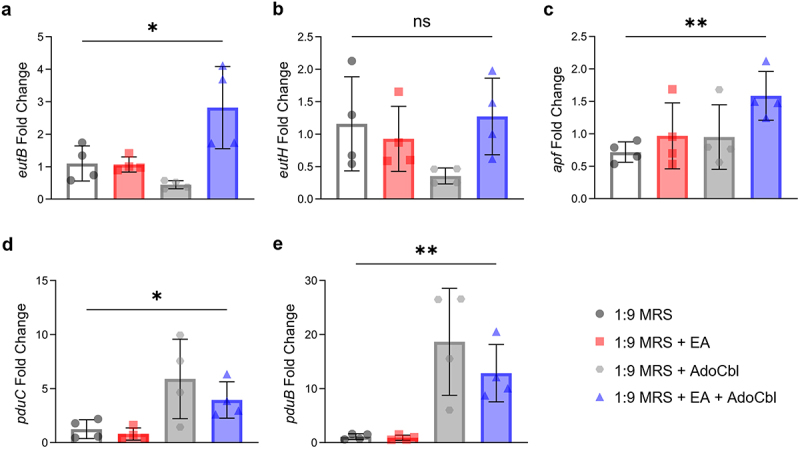
qPCR analysis of *L. brevis* ATCC 14869 *eut*, *pdu*, and *apf* genes.

Differentially expressed genes were defined as those with a |log FC| > 1 and *p* < .05. This analysis revealed 116 differentially expressed genes between cultures grown with and without ethanolamine and adenosylcobalamin (Figure 5a–b). This includes multiple genes from both the *eut* and *pdu* operons (Figure 5e–f), as well as a putative aggregation promoting factor (*apf*) (Table 1). In contrast, growth with ethanolamine or adenosylcobalamin alone resulted in 14 or 0 differentially expressed genes, respectively (Figure 5c–d). qPCR was used to verify the observed expression pattern of select genes of interest, namely, the *eutB*, *eutH*, *pduC*, *pduB* and *apf* genes (Figure 6). In comparison to cultures grown in media alone, there was a significant increase in the expression of *eutB* (two-tailed *t* test; *p* = .0465; Figure 6a), *apf* (two-tailed *t* test; *p* = .0054; Figure 6c), *pduC* (two-tailed *t* test; *p* = .0290; Figure 6d) and *pduB* (two-tailed *t* test; *p* = .0045; Figure 6e) in cultures grown with ethanolamine and adenosylcobalamin relative to the housekeeping genes *gyrA* and *rpoD*.^19,20^ Conversely, there was no significant difference in the expression of *eutH* (two-tailed *t* test; *p* = .8165; Figure 6b).

Cultures of *L. brevis* ATCC 14869 were grown in 1:9 diluted MRS with or without 10 mM EA and 150 nM AdoCbl prior to RNA extraction and sequencing. (a) Venn diagram of differentially expressed genes. The number within each circle represents the number of differentially expressed genes between the different conditions relative to the control. (b–d) Volcano plots of RNA sequencing analysis comparing cultures grown in 1:9 diluted MRS with those grown with EA, AdoCbl, or both metabolites. Data points in green represent significantly upregulated genes, whereas those in red and black signify those significantly downregulated or unchanged, respectively. (e–f) Schematic representation of the *eut* and *pdu* operons found in *L. brevis*. Genes shown in green were found to be significantly upregulated via RNA sequencing analysis in cultures grown with EA and AdoCbl compared to the control, whereas those indicated in black were unchanged.

qPCR was conducted during the stationary growth phase to assess the impact of ethanolamine (EA) on select *L. brevis* ATCC 14869 *eut* (a–b), *apf* (c) and *pdu* (d–e) gene expression. Cultures of *L. brevis* ATCC 14869 were grown in 1:9 diluted MRS with or without 10 mM EA and 150 nM adenosylcobalamin (AdoCbl). Gene expression data points are displayed as mean fold change (relative to *gyrA* and *rpoD*). Error bars represent mean ± standard deviation (SD) of 4 biological replicates (unpaired, two-tailed *t* test). *, *P* < .05; **, *P* < .01; ns = not significant.

**Table 1.** Differentially expressed transcripts by *L. brevis* in the presence of ethanolamine and adenosylcobalamin.

Locus Tag	Gene	Protein Function
FC61_GL000703	*pduU/eutS*	Bacterial microcompartment shell protein
FC61_GL000705	*pduQ/eutG*	Iron-containing alcohol dehydrogenase
FC61_GL000706	*pduP/eutE*	Aldehyde dehydrogenase
FC61_GL000708	*pduO*	Corrinoid adenosyltransferase
FC61_GL000709	*pduN/eutN*	Bacterial microcompartment shell protein
FC61_GL000710	*pduM*	Bacterial microcompartment shell protein
FC61_GL000712	*pduJ*	Bacterial microcompartment shell protein
FC61_GL000713	*pduK*	Bacterial microcompartment shell protein
FC61_GL000714	*pduH*	Diol dehydratase reactivating factor small subunit
FC61_GL000715	*pduG*	Diol dehydratase reactivating factor large subunit
FC61_GL000716	*pduE*	Diol dehydratase small subunit
FC61_GL000717	*pduD*	Diol dehydratase medium subunit
FC61_GL000718	*pduC*	Diol dehydratase large subunit
FC61_GL000719	*pduB*	Bacterial microcompartment shell protein
FC61_GL000720	*pduA*	Bacterial microcompartment shell protein
FC61_GL001105	*eutC*	Ethanolamine ammonia-lyase small subunit
FC61_GL001106	*eutB*	Ethanolamine ammonia-lyase large subunit
FC61_GL001107	*eutA*	Ethanolamine ammonia-lyase reactivating factor
FC61_GL001108	*eutW*	Histidine kinase
FC61_GL001109	*eutV*	Response regulator
FC61_GL001110	*eutG*	Iron-containing alcohol dehydrogenase
FC61_GL001111	*eutT*	ATP cob(I)alamin adenosyltransferase
FC61_GL001634	*apf*	Aggregation promoting factor surface protein

### The cell surface hydrophobicity of *L.* *brevis* ATCC 14869 does not change in the presence of ethanolamine

**Figure 7.**
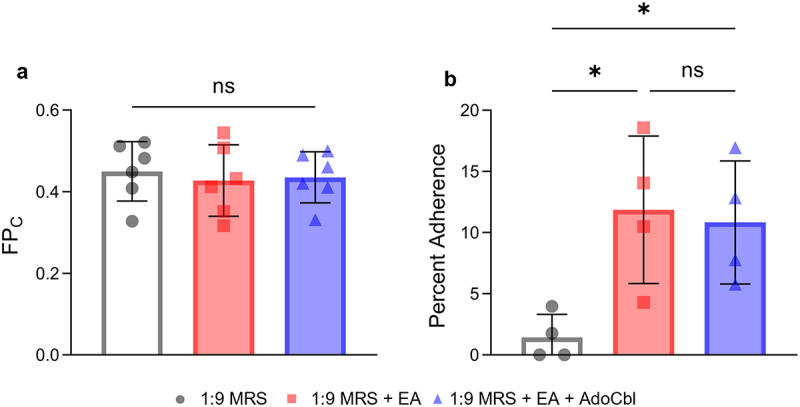
The influence of ethanolamine (EA) on *L. brevis* ATCC 14869 adhesion to abiotic factors.

A MATH assay was used to determine if ethanolamine alters cell surface hydrophobicity; *L. brevis* was grown with or without 10 mM ethanolamine and 150 nM adenosylcobalamin. When differences in the fraction partitioned to the hydrocarbon phase (FP_C_) between groups were assessed, there was no significant difference observed (ANOVA; *F* = 1.387, *p* = .8716; Figure 7a).

(a) A MATH assay was performed using *L. brevis* ATCC 14869 grown in 1:9 diluted MRS with or without 10 mM EA and 150 nM adenosylcobalamin (AboCbl). Adhesion of bacteria to hydrocarbons was evaluated as the fraction partitioned to the hydrocarbon phase (FP_C_), calculated relative to optical density measured at 600 nm. FP_C_ was estimated as final absorbance over initial absorbance (A_f_ /A_o_) of the bacterial suspension. Error bars represent the mean ± standard deviation (SD) of 6 biological replicates (one-way ANOVA). (b) In vitro adhesion to mucin was assessed using a plate-based assay. Percent adherence was calculated relative to the initial CFU/mL quantified prior to adhesion from overnight cultures grown in each respective condition. Quantity of recovered bacteria was calculated through quantification of CFU/mL after a 1-hour incubation followed by washing off non-adherent bacteria. Error bars represent mean ± standard deviation (SD) for 4 biological replicates (one-way ANOVA complemented with Tukey’s multiple-comparison test). *, *P* < .05; ns = not significant.

### The ability of *L.* *brevis* ATCC 14869 to adhere to mucin in vitro increases in the presence of ethanolamine

Using a plate-based assay,^21^ the percent adherence for each well was measured relative to the initial number of bacteria added. There was a significant difference in the percent adherence of *L. brevis* observed between conditions (ANOVA; *F* = 6.061, *p* = .0215; Figure 7b). Cells grown overnight in the presence of ethanolamine (Tukey’s multiple-comparisons test; *p* = .0282) or ethanolamine and adenosylcobalamin (Tukey’s multiple-comparisons test; *p* = .0459) adhered significantly more to mucin-coated wells when compared to those grown in media alone.

### Ethanolamine and adenosylcobalamin influence the ability of *L.* *brevis* ATCC 14869 to adhere to intestinal epithelial cells

**Figure 8.**
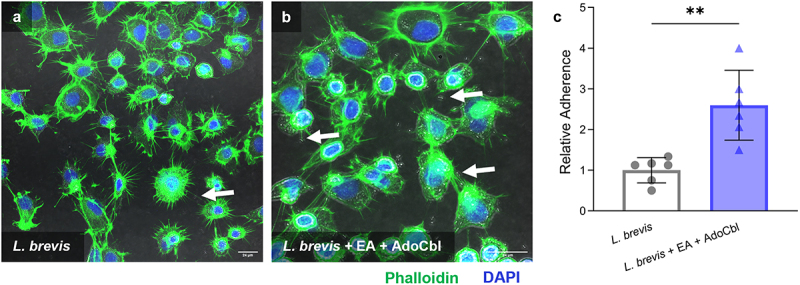
Ethanolamine (EA) influences *L. brevis* ATCC 14869 adhesion to human intestinal epithelial cells.

Caco-2 cells are commonly used as a model of the intestinal epithelial barrier.^22^ Therefore, the ability of *L. brevis* to adhere to Caco-2 cells following growth under various conditions was used as a measure of cellular adherence to the intestinal epithelium. Briefly, bacterial cells grown overnight with or without 10 mM ethanolamine and 150 nM adenosylcobalamin were allowed to adhere to Caco-2 cells and the resulting adherent bacteria were quantified via CFU analysis. There was a significant 2.5-fold increase in the cellular adherence to Caco-2 cells of *L. brevis* ATCC 14869 grown overnight in the presence of 10 mM ethanolamine and 150 nM adenosylcobalamin (two-tailed *t* test; *p* = .0016; Figure 8) relative to bacteria grown in media alone.

Caco-2 cells were incubated with bacteria for 3 hours. Relative adherence was calculated in relation to bacteria recovered from cultures that were grown in 1:9 diluted MRS. *L. brevis* was grown with or without 10 mM EA and 150 nM adenosylcobalamin (AdoCbl) and allowed to adhere Caco-2 cells. White arrows indicate adherent bacteria. Scale bars represent 24 µm. Error bars represent mean ± standard deviation (SD) of 6 biological replicates (unpaired, two-tailed *t* test). **, *P* < .01.

### Ethanolamine and adenosylcobalamin enhance the ability of *L.* *brevis* ATCC 14869 to competitively exclude *S.* Typhimurium LT2 from binding intestinal epithelial cells

**Figure 9.**
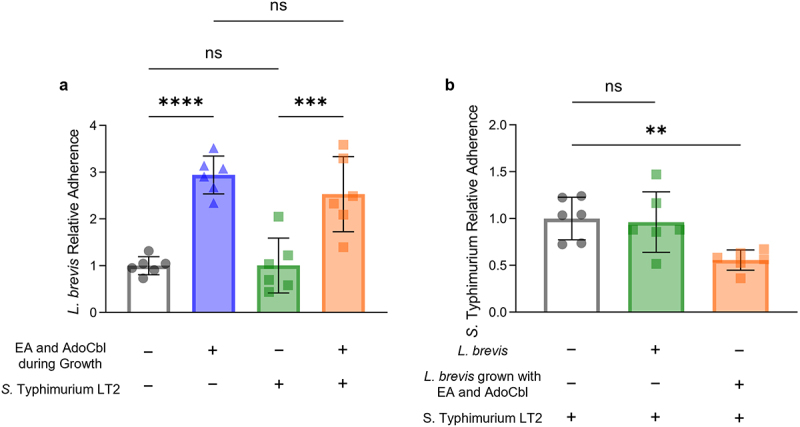
Ethanolamine (EA) enhances the ability of *L. brevis* ATCC 14869 to competitively exclude *Salmonella* Typhimurium LT2 from binding human intestinal epithelial cells.

A competitive exclusion assay was used to assess the ability of *L. brevis* grown with or without 10 mM ethanolamine and 150 nM adenosylcobalamin to competitively exclude *S*. Typhimurium LT2 from binding a model of the intestinal epithelium. There was a significant difference in the relative adherence of *L. brevis* across conditions (ANOVA; *F* = 20.76, *p* < .0001; Figure 9a). In both the presence (Tukey’s multiple-comparisons test; *p* = .0005) and absence (Tukey’s multiple-comparisons test; *p* < .0001) of *S*. Typhimurium LT2, *L. brevis* ATCC 14869 grown with ethanolamine and adenosylcobalamin had significantly increased adhesion relative to cells grown in media alone. Interestingly, the quantity of *S*. Typhimurium LT2 recovered from cells first incubated with *L. brevis* grown with ethanolamine and adenosylcobalamin was significantly less (ANOVA; *F* = 6.485, *p* = .0093) than that recovered from cells incubated with *S*. Typhimurium LT2 alone (Tukey’s multiple-comparisons test; *p* = .0015; Figure 9b).

Caco-2 cells were first incubated with *L. brevis* ATCC 14869 grown overnight with or without 10 mM EA and 150 nM adenosylcobalamin (AdoCbl), washed to remove non-adherent bacteria, and subsequently infected with *S*. Typhimurium LT2 for 30 minutes. Remaining adherent bacteria were recovered and quantified via analysis of CFU/mL. (a) *L. brevis* relative adherence. (b) *S*. Typhimurium LT2 relative adherence. Error bars represent the mean ± standard deviation (SD) of 6 biological replicates (one-way ANOVA complemented with Tukey’s multiple-comparison test). **, *P* < .01; ***, *P* < .001; ****, *P* < .0001; ns = not significant.

## Discussion

This is the first study to demonstrate that a lactic acid bacterium can respond to environmental ethanolamine to alter gene expression and enhance cellular adherence. Despite possessing a fully intact and functional *eut* operon (Figure 4e), *L. brevis* ATCC 14869 does not gain a growth advantage when cultured in the presence of ethanolamine (Figure 1). In fact, despite having increased optical density (Figure 1a), bacterial growth remained unaltered in the presence of physiologically relevant amounts of ethanolamine in standard bacteriological media (Figure 1b). However, *L. brevis* could utilize ethanolamine in low nitrogen conditions at a severe cost to the organisms growth.

**Figure A1.**
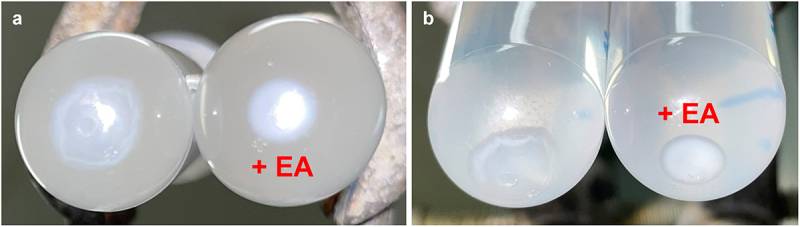
Ethanolamine promotes in vitro aggregation of *Levilactobacillus brevis* ATCC 14869.

While *L. brevis* ATCC 14869 did not display growth benefits from ethanolamine supplementation, it was still plausible that it could preferentially utilize ethanolamine as a carbon or nitrogen source. Upon assessing ethanolamine titers remaining in the culture supernatant following growth, this was not the case (Figure 2b). Unlike *E. coli* CFT073 (Figure 2d), *S*. Typhimurium LT2 (Figure 2h), and *E. faecalis* ATCC 33186 (Figure 2l), *L. brevis* was unable to reduce ethanolamine spiked into the standard culture medium (Figure 2b). Furthermore, while both *E. coli* CFT073 and *S*. Typhimurium LT2 have been shown to use ethanolamine as a signaling molecule to increase the expression of genes related to adhesion,^6^ there were very minimal increases in optical density when grown with ethanolamine in comparison to *L. brevis* ATCC 14869. The same negative result was observed in the Gram-positive bacterium *L. plantarum* ATCC 14917 (Figure 2i), a relative of *L. brevis*. Because the bacteriological media used contains sources of both carbon and nitrogen that may be preferred over ethanolamine, the growth of *L. brevis* was also assessed in both cfMRS and rnMRS. When grown in cfMRS, the same trend was observed where an increased optical density in the presence of ethanolamine was observed but was not accompanied by a reduction of the compound in the supernatant. To our surprise, *L. brevis* significantly reduced ethanolamine from culture when grown in rnMRS, however, ethanolamine utilization dramatically reduced the growth of *L. brevis*. While further investigation is required, it is interesting to speculate that the metabolism of ethanolamine by *L. brevis* leads to the production of toxic metabolites that prevent further growth of the organism. Therefore, these findings suggest that unlike other *eut*-positive pathogens and related lactobacilli, ethanolamine causes significant changes to the phenotype of *L. brevis* ATCC 14869 including an increased propensity to self-aggregate during growth (Figure A1).

Pathogens that possess *eut* genes have a distinct advantage over commensal microbes during the early stages of infection.^23^ EHEC and *S*. Typhimurium LT2 utilize ethanolamine as a nutrient source and as a signal to enhance the expression of virulence genes.^7-10,24^ Using the EutBC ammonia-lyase complex, *eut*-positive pathogens break down ethanolamine into acetaldehyde and ammonia.^23^ These molecules then serve as important carbon and nitrogen sources to enhance bacterial growth in an otherwise competitive gastrointestinal environment.

Although *L*. *brevis* ATCC 14869 could not reduce ethanolamine under most conditions, TEM imaging still revealed the production of microcompartments within the organism (Figure 4d–e). This suggests that the production of microcompartment structures may serve an alternative purpose in this bacterium. Both the ethanolamine and 1,2-propanediol degradative pathways involve aldehyde intermediates that demonstrate cytotoxic activity. Due to this, it has been proposed that microcompartments could be created to sequester these toxic aldehydes and protect the cell.^14,25-27^ In fact, mutants lacking the appropriate microcompartments accumulate high levels of aldehydes that arrested growth.^28,29^ Considering our evidence showing ethanolamine metabolism halts the growth of *L. brevis* (Figure 3b), the formation of microcompartments may be used to limit the escape of these harmful compounds or to prevent ethanolamine metabolism as a whole. In pathogens that possess both *eut* and *pdu* operons, like *Salmonella*, there is a system to repress the transcription of *eut* microcompartment proteins during the expression of *pdu* microcompartment proteins via a PocR-dependent mechanism.^30^ Notably, *L. brevis* ATCC 14869 possesses a fully intact and functional *pdu* operon (Figure 5f) in addition to the *eut* genes (Figure 5e). Our results demonstrated that only the *pdu* shell proteins are expressed upon the activation of both operons (Figure 5), suggesting that *L. brevis* ATCC 14869 might use a similar mechanism to prevent the detrimental mixing of microcompartment proteins.^30^ Therefore, the deleterious effect of ethanolamine utilization on *L. brevis* in rnMRS might be explained by the accumulation of toxic aldehydes resulting from ethanolamine metabolism in the absence of the appropriate microcompartment. In other ethanolamine-utilizing bacteria, like *L. monocytogenes*, the microcompartment-mediated metabolism of ethanolamine is linked to flavin-based extracellular electron transport,^31,32^ which cannot be ruled out for *L. brevis* ATCC 14869. The presence of these structures could also indicate preparation for the metabolism of 1,2-propanediol, which is structurally similar to ethanolamine and whose metabolism is under control of the *pdu* operon.

The ability to form cellular aggregates is a desirable trait for some probiotic microorganisms^33^ because it can mitigate pathogen adherence to the intestinal epithelium by forming a protective barrier through self-aggregation or coaggregation with other commensal bacteria. Indeed, these probiotics may also directly co-aggregate with pathogens to promote clearance in the feces.^34,35^ Therefore, the ability of *L. brevis* ATCC 14869 to enhance aggregation (Figures 1a & 2a) in the presence of ethanolamine is a desirable characteristic, making it a prime candidate for use in probiotic contexts to inhibit pathogen colonization. Previous literature suggests that increased cell surface hydrophobicity of probiotic bacteria improves intestinal colonization and contributes to cellular auto-aggregation.^36,37^ When both ethanolamine and adenosylcobalamin were present, the cell surface hydrophobicity of *L. brevis* ATCC 14869 was not significantly different than cultures grown without ethanolamine (Figure 7a).

Despite our observations, the mechanism that mediates this improved aggregation is still unknown. RNA sequencing analysis revealed that ethanolamine and adenosylcobalamin trigger the upregulation of an aggregation promoting factor-like surface protein (Figure 5 and Table 1). This was confirmed via qPCR analysis, where a significant upregulation in the gene encoding this putative aggregation promoting factor was observed (Figure 6c). Aggregation promoting factors (apf) have been associated with numerous functional roles in lactobacilli.^33^ For instance, *L. coryniformis* DSM 20,001 produces a coaggregation-promoting factor that facilitates binding to pathogenic strains of *E. coli* and campylobacter.^34^ Similarly, an apf synthesized by *L. acidophilus* NCFM was shown to promote an auto-aggregation phenotype as well as the ability to mediate adherence to both intestinal epithelial cells and mucin glycoproteins.^33^ Considering the wealth of literature supporting the functional roles of apf and apf-like proteins in promoting adherence in the gut, the upregulation of an apf by *L. brevis* ATCC 14869 in the presence of ethanolamine and adenosylcobalamin (Figures 5 & 6) to promote adherence (Figures 7b & 8) is not unlikely. This provides a potential mechanism by which this commensal microorganism uses ethanolamine to improve gut epithelial binding and colonization.

While improved adhesion in the presence of ethanolamine is significant, the use of *L. brevis* ATCC 14869 as a probiotic may rely on its ability to prevent pathogen adherence in the gut. Interestingly, *L. brevis* ATCC 14869 retained its increased binding capacity when grown in the presence of ethanolamine and adenosylcobalamin when *S*. Typhimurium LT2 was present in culture (Figure 9a). More importantly, the amount of adherent *S*. Typhimurium LT2 recovered from cells pre-incubated with *L. brevis* ATCC 14869 (grown overnight with ethanolamine and adenosylcobalamin) was approximately half of that recovered from cells incubated with the pathogen alone or those pre-incubated with *L. brevis* ATCC 14869 grown in the absence of ethanolamine. These findings suggest that the production of an apf and the self-aggregation phenotype of *L. brevis* ATCC 14869 resulting from growth in the presence of ethanolamine and adenosylcobalamin can potentially inhibit pathogen binding in the gut. Thus, in vivo studies are warranted to assess both the efficacy and safety of *L. brevis* ATCC 14869 supplementation in reducing both pathogen load and infection severity through competitive exclusion.^38,39^

Ethanolamine reaches its highest bodily concentration (~5 mM) in the gut.^2^ For this reason, it is logical that pathogens, commensals, and probiotic strains possess a mechanism to use this molecule. Although numerous studies have shown that EutR is directly responsible for the upregulation of virulence genes outside the *eut* operon,^6,8^ some virulence genes are still upregulated in ∆*eutR* strains of EHEC.^8^ This suggests that *eut*-positive microbes may possess a currently uncharacterized mechanism, independent of EutR, by which they are able to upregulate specific genes in the presence of ethanolamine. Since *L. brevis* ATCC 14869 does not possess the EutR system, the results of this study implicate the existence of a novel mechanism to sense ethanolamine and regulate gene expression.

In conclusion, the present study has identified a potentially novel apf that is upregulated in *L. brevis* ATCC 14869 in response to ethanolamine and adenosylcobalamin, which mediates adhesion to mucin and intestinal epithelial cells. We have also shown that while *L. brevis* ATCC 14869 can be swayed to utilize ethanolamine in a low nitrogen environment, it is detrimental to the growth of this bacterium.

## Materials and methods

### Chemicals

Ethanolamine hydrochloride was obtained from Sigma-Aldrich® (catalog no. E6133) and stored under the conditions defined by the manufacturer. Appropriate concentrations were made the day of experimental use in De Man, Rogosa and Sharpe (MRS) broth (BD Difco™; catalog no. DF0881-17-5). Adenosylcobalamin was obtained from Sigma-Aldrich® (catalog no. V6629) and was stored at a concentration of 0.5 mM at −20°C in a mixture of ethanol and ddH_2_O (60:40 [v/v]). Dilutions were made and filter sterilized on the day of experimental use in MRS broth from this stock. *p*-Toluenesulfonyl chloride was obtained from Sigma-Aldrich® (catalog no. V001581) and stored under the conditions defined by the manufacturer. The appropriate dilution of *p*-toluenesulfonyl chloride was made in acetonitrile on the day of each experiment.

### Overnight culture preparation

*Levilactobacillus brevis* ATCC 14869 and *Lactiplantibacillus plantarum* subsp. plantarum ATCC 14917 (a *eut*-negative control) were streaked from a frozen stock onto MRS agar and incubated in 5% CO_2_ at 37°C under stationary conditions for 48 and 24 hours, respectively. Experimental cultures were prepared by inoculating 10 mL of MRS broth with a single colony and incubating in 5% CO_2_ at 37°C under stationary conditions for 24 hours.

Uropathogenic isolate *Escherichia coli* CFT073, *Escherichia coli* CFT073 Δ*eutR*,^12^ and *Salmonella enterica* ssp. enterica serovar Typhimurium LT2 were streaked from frozen stocks onto lysogeny broth agar (10 g tryptone, 5 g yeast extract and 10 g NaCl with 15 g agar dissolved in 1 L ddH_2_O; LB) and were incubated aerobically at 37°C under stationary conditions for 24 hours. Experimental cultures were prepared by inoculating 10 mL of LB broth with a single colony and incubating aerobically at 37°C under stationary conditions for 24 hours.

*Enterococcus faecalis* ATCC 33186 were streaked from a frozen stock onto brain heart infusion (BD Difco™; catalog no. DF0037-17-8; BHI) agar and incubated aerobically at 37°C under stationary conditions for 24 hours. Experimental cultures were prepared by inoculating 10 mL of BHI broth with a single colony and incubating aerobically at 37°C under stationary conditions for 24 hours.

### Ethanolamine tolerance assay

Four independent overnight cultures of *L. brevis* ATCC 14869 were prepared as described. Bacterial cultures were then washed twice and resuspended in 10 mL of 1× phosphate-buffered saline (8 g NaCl, 0.2 g KCl, 1.44 g Na_2_HPO_4_ and 0.24 g KH_2_PO_4_ dissolved in 1 L ddH_2_O; pH 7.4; PBS). Exactly 100 μL of the washed bacterial cells were used to inoculate 10 mL of filter-sterilized, 1:9 diluted MRS containing 150 nM adenosylcobalamin and either 0 mM, 5 mM, 10 mM, 50 mM, 100 mM or 500 mM of ethanolamine. 300 μL of the resulting cultures were immediately transferred into a 96-well U-shaped-bottom plate (Falcon; catalog no. 35177) in technical duplicate for growth analysis. Plates were incubated at 37°C for 24 hours and the optical density was measured at 600 nm (OD_600_) every 30 minutes using a microplate reader following a 2 second orbital shake (BioTek, Eon). Final colony forming units per milliliter (CFU/mL) was quantified using MRS plates after 24 hours.

### Ethanolamine clearance assay

Overnight cultures of *L. brevis* ATCC 14869, *L. plantarum* ATCC 14917, *E. coli* CFT073, *E. coli* CFT073 Δ*eutR, S*. Typhimurium LT2, and *E. faecalis* ATCC 33186 were washed and resuspended in PBS and 100 μL was used to inoculate 10 mL of filter-sterilized, 1:9 diluted MRS with or without 10 mM ethanolamine and 150 nM adenosylcobalamin. Cultures of *L. brevis* ATCC 14869 and *L. plantarum* ATCC 14917 were incubated in 5% CO_2_ at 37°C under stationary conditions for 24 hours, while *E. coli* CFT073, *E. coli* CFT073 Δ*eutR, S*. Typhimurium LT2 and *E. faecalis* ATCC 33186 were incubated aerobically at 37°C under stationary conditions for 24 hours. In addition, 300 μL of the resulting cultures were immediately transferred into a 96-well plate for OD_600_ analysis, as described above. After 24 hours, the culture supernatants were removed, and remaining ethanolamine was quantified using high-performance liquid chromatography (HPLC). To assess whether *L. brevis* ATCC 14869 can utilize ethanolamine in the absence of traditional nutrient sources, the same experiments were performed in carbon free MRS^40^ (5 g peptone, 5 g meat extract, 2.5 g yeast extract, 1 g K_2_HPO_4_, 0.5 g polysorbate 80, 2.5 g C₂H₃NaO₂, 1 g C_6_H_17_N_3_O_7_, 0.2 g MgSO_4_·7H2O, and 0.05 g MnSO4·H2O dissolved in 400 mL ddH_2_O; pH 6.7; cfMRS), reduced nitrogen MRS (1 g peptone, 0.8 g meat extract, 0.4 g yeast extract, 20 g D-Glucose, 2 g K_2_HPO_4_, 1 g polysorbate 80, 5 g C₂H₃NaO₂, 0.2 g C_6_H_17_N_3_O_7_, 0.2 g MgSO_4_·7H2O, and 0.05 g MnSO4·H2O dissolved in 1 L ddH_2_O; pH 6.5; rnMRS), and nitrogen free MRS (20 g D-Glucose, 2 g K_2_HPO_4_, 1 g polysorbate 80, 5 g C₂H₃NaO₂, 0.2 g MgSO_4_·7H2O, and MnSO4·H2O dissolved in 1 L ddH_2_O; pH 6.5; nfMRS). A reduced carbon MRS group was not included as 1:9 diluted MRS serves this purpose.

Prior to HPLC analysis, samples and ethanolamine standards were subject to a previously described derivatization protocol using *p*-toluenesulfonyl chloride.^41^ Briefly, the culture supernatant was diluted (1:9) with HPLC grade H_2_O and combined with 0.5 M potassium phosphate buffer (pH 11) and 10 mg/mL *p*-toluenesulfonyl chloride (dissolved in HPLC grade acetonitrile) at a ratio of 1:1:2. This reaction mixture was vortexed and incubated at 56°C for 30 to 45 minutes. The samples were subsequently vortexed and filtered (0.45 μM) into amber HPLC vials. An Agilent 1100 HPLC instrument equipped with a degasser (G1379A), quaternary pump (G1311A), autosampler (G1313A) and diode array detector (G1315B) was used for ethanolamine quantification of all the samples and standards. The samples were separated using an Agilent Poroshell 120 EC-C18 (4.6- by 150-mm i.d., 4-μm particle size) column preserved at ambient temperature and detected using a diode array detector at 228 nm. The mobile phase was comprised of acetonitrile and water (60:40 [v/v]) spiked with 0.1% (v/v) orthophosphoric acid. An injection volume of 10 μL at a flow rate of 0.85 mL/min was used. Run times were 12 minutes long, with ethanolamine eluting at ~2.7 minutes. Data were analyzed using ChemStation B.04.03 with reference to an external calibration curve (1 to 10 mM) for ethanolamine quantification.

### TEM of bacterial cells

TEM imaging was performed at the Advanced Analysis Centre at the University of Guelph (Guelph, Ontario, Canada). Briefly, overnight cultures of *L. brevis* ATCC 14869 were grown in 40 mL of filter-sterilized, 1:9 diluted MRS with or without 10 mM ethanolamine and 150 nM adenosylcobalamin by inoculating it with 100 μL of washed bacterial cells and incubating overnight, as previously described above. Subsequently, cultures were centrifuged for 10 minutes at 5000 × *g*. Bacterial pellets were then washed and re-pelleted in filter-sterilized 20 mM HEPES (pH 6.8) prior to sample preparation. Bacterial cells were fixed with 2.5% (w/v) glutaraldehyde and 2% (w/v) paraformaldehyde in 50 mM HEPES, enrobed in Noble agar and fixed with 1% (w/v) osmium tetraoxide before being embedded in LR White resin. 80 nm sections were cut and then contrast stained with both lead citrate and uranyl acetate. Sections were then imaged using a Tecnai F20 G2 transmission electron microscope operated at 120 kVe equipped with a Gatan Ultra scan 4K CCD camera under standard operating conditions.

### RNA extraction and reverse transcription

Overnight cultures of *L. brevis* ATCC 14869 at stationary phase were grown in 40 mL of filter-sterilized, 1:9 diluted MRS with or without 10 mM ethanolamine and 150 nM adenosylcobalamin were centrifuged at 4°C for 5 minutes at 6000 × *g* and the bacterial pellet was resuspended in 1 mL of cold TRIzol reagent (Ambion; catalog no. 15596018) for 10 minutes at room temperature. Next, the bacteria and TRIzol solution was transferred to bead-beating tubes containing approximately 100 μL of 0.1 micron zirconium beads. These tubes were then incubated for 5 minutes at room temperature, and subsequently placed in a Bead Beater (MRC) set at 7 m/s for two cycles of 45 seconds. Following this, 0.2 volumes of chloroform (ThermoFisher) were added, and the samples were briefly vortexed prior to 10 minutes of incubation at room temperature. The samples were then centrifuged at 4°C for 20 minutes at 13,000 rpm. The aqueous layer containing the RNA was then removed and mixed with an equal volume of 70% ethanol prepared using nuclease-free water. Then, 600 μL of the resulting solution was transferred to a spin column and collecting tube (ThermoFisher; catalog no. 12183025) and centrifuged at 12,000 × *g* for 1 minute at room temperature. This process was repeated until the entire sample volume was spun through the cartridge. The flow-through was subsequently discarded, and 600 μL of Wash Buffer I (ThermoFisher; catalog no. 12183025) was added to the spin cartridge. The cartridge was then centrifuged at 12,000 × *g* for 1 minute at room temperature and the flow-through was discarded. The spin cartridge was subsequently inserted into a new collection tube. Next, 80 μL of preprepared PureLink® DNase mixture (ThermoFisher; catalog no. 12185010) was added directly onto the surface of the spin column and allowed to incubate at room temperature for 15 minutes. Then, 600 μL of Wash Buffer I was again added to the spin cartridge and centrifuged at 12,000 × *g* for 1 minute at room temperature. The flow-through was then discarded, and the spin cartridge was again placed in a new collection tube. Next, 600 μL of Wash Buffer II (ThermoFisher; catalog no. 12183025) was added to the spin cartridge and centrifuged at 12,000 × *g* for 1 minute at room temperature. The flow-through was discarded, and this step was repeated once. After discarding the flow-through for a second time, the spin cartridge was centrifuged again at 12,000 × *g* for 2 minutes to dry the membrane. The spin cartridge was then placed in a new microfuge tube, and 50 μL of prewarmed (56°C) nuclease-free water was added to the center of the cartridge. The cartridge was then centrifuged at 12,000 × *g* for 2 minutes to elute the RNA. The resulting solution was then separated into preferred aliquots for quality and quantity analysis, qPCR and RNA sequencing.

After RNA preparation, cDNA was synthesized from 1000 ng of RNA samples aliquoted for qPCR using a High-Capacity cDNA reverse transcription kit (Applied Biosystems; catalog no. 4368813) as per the manufacturer’s instructions.

### RNA sequencing analysis

RNA was extracted according to the previously described method, and samples were assessed for quality and quantity using an Agilent 2100 Bioanalyzer. All samples demonstrated a high RNA Integrity Number (RIN) ≥ 9. To prepare the libraries for RNA sequencing analysis, the Stranded Total RNA Prep Ligation with Ribo-Zero Plus kit from Illumina and 10 bp IDT for Illumina indices were used. A NextSeq2000 system was employed for sequencing, producing high-quality reads of 2 × 51 bp.

Quality control and adapter trimming was performed using bcl2fastq (v2.20.0.445). Read mapping was done with HISAT2 (v2.2.0)^42^ and read quantification was performed using Subread’s featureCounts functionality (v2.0.1).^43^ The read counts were then loaded into R and normalized using the Trimmed Mean of *M* values (TMM) algorithm in edgeR (1.14.5).^44^ Subsequent values were converted to counts per million (cpm). Differential expression analysis was then performed using edgeR’s exact test for differences between two groups of negative-binomial counts with an estimated dispersion value of 0.1. Furthermore, the qlfTest was conducted for all genes; differentially expressed genes were identified as those with a |log Fold Change| > 1 and *p* < .05.

### qPCR analysis

RNA was extracted and reverse transcription of *L. brevis* ATCC 14869 samples was performed as previously described. The prepared cDNA was diluted 15× in nuclease-free water and used for the qPCR reaction with the PowerTrack™ SYBR Green Master Mix (ThemoFisher; catalog no. A46113). The primers used in this study, designed using Primer-BLAST,^45^ are described in Table A1. In summary, *gyrA* and *rpoD* were used as reference genes and the primers targeting *eut* operon (*eutB*, *eutH*), *pdu* operon (*pduC*, *pduB*) and the *apf gene* were used as indicators of *eut* operon, *pdu* operon, and *apf* gene expression. Reagent volumes for the 20 μL reactions contained 5 μL of diluted cDNA, 10 μL of the PowerTrack™ Master Mix, and 5 μL of primer mix containing 2 μM of each the forward and reverse primers (final concentration of 500 nM). Each reaction was performed in technical triplicate and qPCR was conducted on a QuantStudio 5 real-time PCR system and analyzed using the QuantStudio Design and Analysis software v1.4.3 (ThermoFisher). Reaction conditions were as follows: 50°C for 2 min, 95°C for 10 min, 40 cycles of 95°C for 15 s and 60°C for 1 min. The fold change of each target gene was determined through comparison with the average expression of the reference genes (2^−ΔΔCT^).^46^

### Microbial adhesion to hydrocarbons (MATH) assay

Overnight cultures of *L. brevis* ATCC 14869 were grown in 10 mL of filter-sterilized, 1:9 diluted MRS with or without 10 mM ethanolamine and 150 nM adenosylcobalamin. These cultures were centrifuged at 5000 × *g* for 15 minutes and resuspended in 10 mL of ddH_2_O containing 0.2 mM NaCl (pH 6). A microplate reader (BioTek, Eon) was used to calculate the initial absorbance (A_o_) at 600 nm of each sample. A 5 mL aliquot of these washed bacteria cells was then mixed with 300 μL of n-hexadecane (Sigma Aldrich®; catalog no. H6703) and vortexed for 2 minutes. The samples were allowed to rest for 15 minutes to facilitate phase separation. Next, a sample of the bacterial suspension was retrieved and dispensed into a new tube. The absorbance (A_f_) at 600 nm of the recovered sample was then calculated. To determine the fraction partitioned to the hydrocarbon phase (FP_c_), the following formula was used:

$$FP_{c}=1-A_{f}/A_{o}$$

### In vitro mucin adhesion assay

A mucin adhesion assay^21^ was performed in 96-well U-shaped-bottom plates (Falcon; catalog no. 35177). Initially, 100 μL of a mucin solution (10 mg/mL) in 1× PBS was immobilized in each well for 1 hour at 37°C. This was followed by overnight incubation at 4°C. The wells were then washed with 200 μL of 1× PBS and incubated with bovine serum albumin (20 g/L; BSA [Sigma Aldrich®; catalog no. A4628]) for 2 hours at 4°C before being washed again with PBS to eliminate the unbound BSA. Overnight cultures of *L. brevis* ATCC 14869 were grown in filter-sterilized, 1:9 diluted MRS with or without 10 mM ethanolamine and 150 nM adenosylcobalamin alongside the 96-well plate preparation. The initial CFU/mL was determined for each overnight culture. Next, 100 μL of each culture was added to the wells and incubated aerobically for 1 hour at 37°C. After incubation, the wells were washed five times with 200 μL of sterile citrate buffer (8.3 g/L NaCl, 0.26 g/L KCl, and 1.5 g/L Na_2_HPO_4_, 1 M citrate; pH 5) to remove the unbound bacteria. Lastly, 200 μL of 0.5% (v/v) Triton X-100 (Sigma Aldrich®; catalog no. T8787) in PBS was added to remove adherent bacteria and the contents of each well were thoroughly mixed and plated to obtain final CFU/mL. Percent adherence was calculated relative to the initial CFU/mL determined prior to adhesion.

### Caco-2 bacterial adhesion assay

Caco-2 adhesion assays and confocal imaging were performed in 24-well plates (Sarstedt; catalog no. 83.3922). Caco-2 cells were grown in an incubator at 37°C with 5% CO_2_ and 95% relative humidity to ~80–90% confluence in Minimum Essential Medium (Gibco™; catalog no. 2416804; MEM) containing 20% (v/v) fetal bovine serum (Gibco™; catalog no. 12483020; FBS) and 2 mM L-glutamine (Sigma Aldrich®; catalog no. G3126). On the day of experimentation, the cells were washed twice with PBS and subsequently placed in MEM prior to infection. Entire experiments were performed in technical duplicate in two identical plates, with one being used for CFU/mL analysis and the other for confocal imaging purposes. Overnight cultures of *L. brevis* ATCC 14869 were grown in advance in filter-sterilized, 1:9 diluted MRS with or without 10 mM ethanolamine and 150 nM adenosylcobalamin. The bacterial cells were centrifuged at 5000 × *g* for 10 minutes and resuspended in PBS. These cultures were then used to infect the wells containing Caco-2 cells (ATCC; HTB-37™). The cells were then incubated for 3 hours at 37°C in 5% CO_2_. Each well was washed thrice with PBS to remove all nonadherent bacteria. To calculate remaining CFU/mL of adherent bacteria, 0.4% (v/v) Triton X-100 in PBS was used to induce lysis of the Caco-2 cells. The contents of each well were thoroughly mixed, and the resulting suspension was used to calculate remaining CFU/mL. Relative adherence was calculated in relation to the adherent bacteria recovered from the cultures grown without ethanolamine or adenosylcobalamin. For imaging, a Nikon A1R confocal microscope was used. Prior to imaging, the wells were stained with 30 mM of Phalloidin for 30 minutes and 5 nM of DAPI for 5 minutes and were subsequently submerged in Prolong® Gold Antifade Reagent (ThermoFisher; catalog no. P36930). Images were acquired using both DIC and fluorescent modes, and subsequently overlayed using the Nikon A1R software to view multiple channels simultaneously.

### Caco-2 competitive exclusion assay

Caco-2 competitive exclusion assays were performed in 24-well plates. Caco-2 cells were grown in an incubator at 37°C with 5% CO_2_ and 95% relative humidity to ~80–90% confluence in MEM containing 20% (v/v) FBS and 2 mM L-glutamine. On the day of experimentation, the cells were washed twice with PBS and subsequently placed in 1 mL of MEM. Overnight cultures of *L. brevis* ATCC 14869 were grown in filter-sterilized, 1:9 diluted MRS with or without 10 mM ethanolamine and 150 nM adenosylcobalamin. Overnight cultures of *S*. Typhimurium LT2 were grown in LB. Overnight cultures of *L. brevis* and *S*. Typhimurium contained approximately 1 × 10^8^ CFU/mL. The bacterial cells were centrifuged at 5000 × *g* for 10 minutes and resuspended in PBS. Next, Caco-2 cells were incubated with or without *L. brevis* ATCC 14869, which was grown overnight with or without ethanolamine and adenosylcobalamin, for 3 hours at 37°C in 5% CO_2_. Each well was subsequently washed with PBS to remove non-adhered bacteria. Cells were then placed in MEM before being infected with *S*. Typhimurium LT2 for 30 minutes under the same incubation conditions. Once again, each well was washed thrice with PBS to remove any unadhered bacteria. To calculate remaining CFU/mL of adherent bacteria, 0.4% (v/v) Triton-X100 was used to lyse the Caco-2 cells. The contents of each well were thoroughly mixed, and the resulting suspension was plated on both MRS and LB agar to calculate remaining CFU/mL of *L. brevis* and *S*. Typhimurium LT2, respectively. For *L. brevis* ATCC 14869, relative adherence was calculated in relation to the adherent bacteria recovered from the cultures grown without ethanolamine or adenosylcobalamin and incubated with the Caco-2 cells in MEM alone. For *S*. Typhimurium, relative adherence was similarly calculated in relation to bacteria recovered from wells incubated with *S*. Typhimurium alone.

### Statistical analyses

GraphPad Prism v9.5.1 was used to perform most statistical analyses. The Shapiro–Wilk test or D’Agostino and Pearson test was used to assess normality. Normally distributed data were compared with an unpaired, two-tailed *t* test. One-way ANOVA was used for comparison of three or more independent groups, complemented with Tukey’s multiple-comparisons test. Nonparametric data were compared with an unpaired Mann–Whitney test or an unpaired, one-way Kruskal–Wallis test, complemented with Dunn’s multiple-comparison test. R v4.2.1 was used to analyze RNA sequencing data.

## Figures and Tables

Cultures of *L. brevis* were grown in 1:9 diluted MRS with 150 nM adenosylcobalamin (left) or both 150 nM adenosylcobalamin and 10 mM ethanolamine (right), as previously described. After 24 hours of growth, bacterial culture tubes were imaged to qualitatively visualize cellular aggregation.

## Data Availability

Illumina RNA sequencing reads can be found in NCBI under BioProject accession number PRJNA1001957.

## Appendix

**Table A1.** *Levilactobacillus brevis* ATCC 14869 qPCR primer sequences.

Gene Target (Locus Tag)	Primer Sequence
*eutB* (FC61_GL001106)	F: 5’ TTGCAGACGGGATGAGAACG 3’ R: 5’ TCGTCGCGGATTGACATCAG 3’
*eutH* (FC61_GL001096)	F: 5’ TGGCCACTAATCCAGCAGC 3’ R: 5’ CAGCATTGCGATGACGTTGG 3’
*pduC* (FC61_GL000718)	F: 5’ GAACGCTTGGTCATTGGCAG 3’ R: 5’ GGTATTCGCTCCGTCTTGGG 3’
*pduB* (FC61_GL000719)	F: 5’ ACACCAGCCGTCTTAATGGC 3’ R: 5’ GACGTGTATAACTCCGCCGC 3’
*apf* (FC61_GL001634)	F: 5’ GTGACGTCTGTGGTTACGCC 3’ R: 5’ AGTAGCGACCATTTCGTGCG 3’
*gyrA* (FC61_GL001235)	F: 5’ GACTGGGATGCGGATTGTGA 3’ R: 5’ CCGGCGGATAACTTCCAACT 3’
*rpoD* (FC61_GL000324)	F: 5’ AGCGTGGTTGATGCTAAGGG 3’ R: 5’ ATGCGACTTCTTCATCGGCA 3’
